# Investigating functional impairment as a mediator of the association between internalising symptoms and low wellbeing

**DOI:** 10.1016/j.jad.2026.121517

**Published:** 2026-02-26

**Authors:** Tanika R. Sgherza, Rodrigo Becerra, Lauren M. Bylsma, David M. Fresco, Kristin Naragon-Gainey

**Affiliations:** a School of Psychological Science, University of Western Australia, Crawley, WA, Australia; b Departments of Psychiatry and Psychology, University of Pittsburgh, Pittsburgh, PA, USA; c Department of Psychiatry, University of Michigan, Ann Arbor, MI, USA

**Keywords:** Wellbeing, Generalised anxiety, Depression, Internalising symptoms, Functional impairment, Psychosocial functioning

## Abstract

**Objectives:**

There is a lack of focal research on transdiagnostic factors that explain the influence of internalising symptoms on wellbeing. It is plausible that low wellbeing may be a consequence of functional impairment resulting from symptoms, but this has yet to be explored.

**Methods:**

Internalising symptoms (i.e., major depression, persistent depression, generalised anxiety), impairment, and wellbeing were assessed in a community sample (*N* = 408) using semi-structured clinical interviews and self-report measures. Structural equation models were conducted to analyse effects of symptoms on overall wellbeing (concurrently and with wellbeing assessed one month later), mediated by impairment. Exploratory analyses examined the mediating effect of impairment on the relationship of individual depression and anxiety symptoms with wellbeing.

**Results:**

Impairment accounted for a portion of the association between interviewer-rated major depression and generalised anxiety symptoms and wellbeing, but not persistent depression symptoms. The results for anxiety were replicated with self-report measures, but not for major depression. Indirect effects generally persisted over time, but only when not controlling for baseline wellbeing. Exploratory analyses revealed significant indirect effects of individual symptoms of generalised anxiety (uncontrollability, restlessness, irritability, sleep disturbance, fatigue, and troubles concentrating due to worry) and major depression (depressed mood, anhedonia, troubles concentrating, feelings of guilt/blame, and fatigue).

**Conclusion:**

These findings support the hypothesis that impairment associated with internalising symptoms is one factor connecting symptoms, particularly anxiety, to reduced wellbeing, and thus is an important research area and therapeutic target.

## 1. Introduction

Over the last several decades, there has been an increased focus on understanding wellbeing in the context of internalising symptoms (i.e., depression, anxiety, referred to as “symptoms” throughout) (Stein and Heimberg, 2004; Vaingankar et al., 2020; Zadow et al., 2017). Well-being is a multifaceted construct that is separable from symptoms (Magalhães, 2024), and it broadly includes life satisfaction, happiness, sense of meaning and purpose, and positive integration in society (Keyes, 2005). Furthermore, it is well-established that elevated symptoms are moderately associated with reduced wellbeing across all facets (Sgherza et al., 2025), though the association varies in strength across individuals, ranging from weak to strong (Kraiss et al., 2022; Seow et al., 2016; Sgherza et al., 2025; Völker, 2020). Among people with elevated symptoms, about 9–17% maintain moderate to high levels of wellbeing (Magalhães, 2024). However, few studies have assessed underlying mechanisms that connect symptoms to poorer wellbeing, which limits understanding of *how* wellbeing can be preserved for people with elevated symptoms.

A handful of cross-sectional studies have identified mediators of the relationship between various symptoms and facets of wellbeing in specific populations. In college students, low emotional self-efficacy mediated the relationship between social anxiety and subjective well-being (Ye et al., 2021), and emotional perception clarity mediated the relationship depression and single-item life satisfaction (Lee, n.d.). In some medical populations, health-related distress mediated the relationship between depression and psychological wellbeing in people with diabetes (Ajele et al., 2022), and low perceived social support mediated the relationship between anxiety and life satisfaction in pregnant women (Yu et al., 2020). While this provides insight into mediators between specific symptoms and wellbeing facets in specific populations, further research is needed to assess transdiagnostic constructs in general populations, using multidimensional measures and longitudinal designs.

One such transdiagnostic mediator may be functional impairment (also referred to as role functioning specifically or psychosocial functioning broadly; Yang et al., 2022). Functional impairment (referred to as “impairment” in the rest of this paper) is the detrimental impact of symptoms on one’s ability to perform valued roles (e.g., working, studying, socialising), complete activities of daily life (e.g., making meals, basic hygiene, housework), and/or form and maintain relationships (Üstun and Kennedy, 2009; Stein and Heimberg, 2004). Impairment is a key criterion in diagnoses of most mental disorders (DSM–5; American Psychiatric Association, 2013) and is an important treatment target, given the immense individual and societal cost of poor functioning (Beyer and Boazak, 2021; McKnight et al., 2016). Though positively related, impairment is also separable from symptom severity, and it varies within and across community and clinical populations (Kiel et al., 2024; Rodríguez et al., 2012; McKnight and Kashdan, 2009; McKnight et al., 2016). Evidence and theoretical support for impairment as a potential mechanism between symptoms and wellbeing is discussed in the following subsections.

### 1.1. Symptoms and impairment

Observational studies show that depressive symptoms (e.g., symptoms of major depressive disorder (MDD) and persistent depressive disorder (PDD)) have significant moderate negative associations with numerous functioning domains, including home, social, and work life (McKnight and Kashdan, 2009; Fried and Nesse, 2014; Hellerstein et al., 2010). In particular, symptoms of fatigue, poor concentration, anhedonia, and depressed mood may have greater associations with impairment than do suicidality, weight change, and sleep disturbance, assessed cross-sectionally and longitudinally (Tanner et al., 2019; Fried and Nesse, 2014; Xiao et al., 2018). Intervention studies by Malkki et al. (2023) and Vinckier et al. (2017) also showed that improvements in symptoms are associated with reductions in impairment. Similarly, symptoms of generalised anxiety disorder (GAD) are also associated with greater impairment in observational studies, but to a lesser extent than MDD (Kessler et al., 2009; McKnight et al., 2016). Observational cross-sectional studies show that physical symptoms associated with worry (such as feeling keyed up/on edge and concentration difficulties) are most strongly associated with impairment, in part due to reduced productivity (Romera et al., 2011; Tanner et al., 2019).

Theoretically, there are many ways that symptoms may contribute to impairment. Impairment from depression could be due to motivational or social-cognitive deficits, and changes in reward processing (Fervaha et al., 2016; Kraft et al., 2023; Weightman et al., 2019). Additionally, slower information processing speed, cognitive inflexibility, and executive dysfunction make it difficult to perform day-to-day tasks that require attention or adaptation (Cambridge et al., 2018; Freidl et al., 2022; Lopes et al., 2018). Similarly, impairment from GAD symptoms could be attributed to difficulties shifting attention from worry to required tasks, as well as exacerbated perceptions of threat (e.g., at work, school, social settings) which may lead to distress and avoidance (Freidl et al., 2022). Moreover, associated cognitive and emotional difficulties (e.g., emotion dysregulation, negative automatic thoughts) may further contribute to social difficulties and relationship conflict (Freidl et al., 2022; Yang et al., 2024).

### 1.2. Impairment and wellbeing

Like psychological symptoms, wellbeing is also closely intertwined with how one functions in life. Although some types of functioning (e.g., psychosocial functioning, a broader term encapsulating social and subjective psychological functioning) overlap with conceptualisations of wellbeing, there are more distinct conceptualisations. Specifically, in this study, we operationalise impairment as difficulties in role functioning (i.e., the ability to fulfill role expectations in social, work, and other domains), which is considered distinct from wellbeing (see Yang et al., 2022 for a detailed review).

Evidence suggests a small to moderate inverse relationship between impairment (e.g., work, social roles) and hedonic, eudaimonic and social wellbeing across non-clinical samples and clinical samples with mood and anxiety disorders (Asif et al., 2020; Franken et al., 2018). Indicators of role functioning contribute to greater eudaimonic wellbeing (Huppert, 2009) and life satisfaction (Seow et al., 2016), and may act as protective factors of life satisfaction when experiencing symptoms (Grych et al., 2020). Indeed, positive functioning in various domains may facilitate a greater sense of satisfaction with life via increased social support and positive feelings, as well as increased fulfillment and stress-relief (Grouden and Jose, 2015; Mansfield et al., 2020; Santini et al., 2020).

When one’s ability to function adequately becomes impaired due to symptoms, all facets of wellbeing may be compromised (Huppert, 2009). In this case, impairment may disrupt important life domains that otherwise promote wellbeing and resilience. According to Hobfoll’s Conservation of Resources Theory (Hobfoll, 1989, 2001), wellbeing is promoted by the ability to acquire, protect, and conserve resources (e.g., social support, financial stability), and such resources are likely to be negatively impacted by impairment (e.g., problems in or loss of job or relationships). Activity restrictions may also reduce resources to cope with such stressors, opportunities to engage with loved ones and form social connections, and to cultivate positive emotions, which are all important contributors to building and maintaining wellbeing and resilience (Bonanno et al., 2007; Chao, 2014; Goodman et al., 2018; Yang et al., 2022).

As such, it is plausible that low wellbeing may be the result of associated impairment resulting from anxiety and depression symptoms, rather than solely due to the symptoms themselves. That is, in the absence of impairment, the link between symptoms and wellbeing may be weaker. To our knowledge, only one cross-sectional study investigated impairment as a mechanism that explains the relationship between depressive symptoms and a wellbeing-related outcome, *quality of life* (Zhou et al., 2022). Network analysis revealed that self-reported impairment partially mediated the association between individual depressive symptoms and quality of life in a sample of Chinese patients diagnosed with MDD (Zhou et al., 2022). While this initial investigation is promising, the study is limited in that impairment and quality of life were self-reported and cross-sectional, and it is unclear to what extent results may be specific to depression or generalizable to other symptoms (e.g., anxiety).

### 1.3. The current study

To address these gaps, this study tested whether impairment contributes to the inverse relationship between symptoms and wellbeing in a community sample with elevated rates of a broad range of symptoms. The study includes interviewer and self-report assessments of generalised anxiety (referred to as anxiety throughout for brevity),depression symptoms, and impairment, as well as self-reported wellbeing at two timepoints. It was hypothesised that impairment would at least partially mediate the inverse relationship of symptoms of both anxiety and depression with overall wellbeing (emotional, psychological, social), at the same time point and over time. A primary model with interviewer data and a secondary model with self-reported data were examined.^1^ An exploratory aim was to understand which symptoms drive impairment and detriments in wellbeing, as it is likely that symptoms contribute differentially to impairment (Tanner et al., 2019; Zhou et al., 2022).

## 2. Method

We conducted secondary data analysis on a larger dataset, and the research questions, measures, and analyses for the current study were pre-registered on Open Science Framework prior to conducting analyses: https://doi.org.10.17605/OSF.IO/R8WUP. No other published manuscripts from this dataset report this set of variables together.

### 2.1. Participants

Community participants were recruited in Perth, Western Australia from 2020 to 2024, using advertisements on social media, flyers, university websites, and word of mouth. For the larger study, participants were excluded if younger than 18 or older than 65; current mania, psychosis or substance use disorder; pregnancy; cardiovascular condition or medication that impacted heart rate variability. The minimum required sample size (*N* = 350) was determined based on power simulations conducted for the larger study. Our recruitment target was that at least 50% of participants had elevated rumination or worry scores at screening, to increase likely representation of elevated symptoms of depression and anxiety.

The sample consisted of 408 participants aged 18 to 65 (*M* = 34.41, *SD* = 13.73), who were mostly female (60.3%), single (53.4%) and White (64.7%) or Asian (29.4%). Approximately one third of participants were full-time students (36.5%) and/or employed part-time (31.9%), while 20% were unemployed (note multiple selections were possible). Most participants were well-educated (i.e., 58.5% completed a university degree or higher), but there were a wide range of household incomes, with 27.2% earning $10,000–$20,000 and 24.8% earning more than $100,000. Consistent with our recruitment target, over half had elevated scores on rumination and/or worry (55.0%). Most participants (54.7%) had a history of psychological treatment, with 23.5% currently receiving therapy and/or taking medication.

### 2.2. Procedure

The larger study involved three phases, but only measures from Phase 1 and Phase 3 are analysed here. Participants completed an online screening questionnaire (and where necessary, follow up phone interviews) to determine eligibility. Eligible participants were invited to enroll in Phase 1, completing questionnaires from home and attending a 3-to-4-h lab session with a semi-structured clinical interview, as well as psychophysiological measurements and behavioural computer tasks not analysed here. Participants were paid $20 AUD for completion of the baseline survey, and $20 AUD per hour for participation in the baseline lab session. Phase 2 was a 10-day ecological momentary assessment that is not reported in the current study. Phase 3 (follow-up) consisted of completion of brief monthly online surveys for 12 months, commencing one month after the baseline session (only the first month is analysed in the current study). Participants had one week to fill out the online survey once it had been sent out and were paid $10 AUD for each completed survey. As an extra incentive, participants were also entered into a raffle to win an iPad if they completed at least 8 of the 12 surveys. This study was approved by the Human Research Ethics Committee at the University of Western Australia. Participant self-report data were excluded (*N* = 38 at baseline, *N* = 19 at follow-up) if one or more inattention checks were failed (i.e., responding incorrectly to >1 attention check items, average responses to invalidity items [Simms et al., 2011] > 2SDs from the sample mean, and/or speeded responding as defined by Greszki’s (2015) QualiTime index). Missing data was limited (78.7% to 99.8% of data was present across pairs of variables).

### 2.3. Measures

Item overlap across measures of symptoms, wellbeing, and impairment were considered prior to analysis, given that it could be a potential concern. Following consideration, measurement overlap were deemed minor and unlikely to substantially influence results (see online supplement p. 8 for more detail).

#### 2.3.1. Anxiety and depression symptoms

##### 2.3.1.1. Anxiety and Related Disorders Interview Schedule for DSM-5 (ADIS-5) - adult version (Brown and Barlow, 2005).

The ADIS-5 is a semi-structured interview designed to reliably diagnose and dimensionally assess a range of current internalising disorders in adults. This study only assessed current GAD, MDD, and PDD using the original version (see online supplement for item/scoring details). The interviews were conducted and scored by thoroughly trained research assistants and PhD students, and the team regularly met for supervisory discussions of interviews with a clinical psychologist (KN-G). Inter-rater reliability was assessed based on audio recordings of interviews (*n* = 95, 23.34% of the full sample), indicating excellent reliability (intraclass correlations (ICCs) = 0.97–0.98).

##### 2.3.1.2. Inventory of Depression and Anxiety Symptoms – Expanded version (IDAS-II) (Watson et al., 2012).

At baseline, participants rated experiences in the past two weeks, including today, using a 5-point Likert scale from 1 (Not at all) to 5 (Extremely). The current study used the original version, with the Dysphoria subscale (9 items) to assess Depression, and the Anxious Mood subscale (7 items) to assess generalised anxiety. Note that there is one identical item in these scales, so it was removed from Dysphoria to eliminate item overlap. Both scales demonstrated excellent internal consistency (Dysphoria: Cronbach’s α = 0.90, Anxious Mood: Cronbach’s α = 0.93) in this sample, which is consistent with prior college, clinical and community samples (Watson et al., 2012).

#### 2.3.2. Functional impairment

##### 2.3.2.1. ADIS-5 - Adult Version (Brown and Barlow, 2005).

The single-item dimensional rating for impairment (rated 0 to 8) from ADIS-5 was used to reflect interviewer-rated impairment specific to symptoms of each assessed disorder. At the end of each module, participants were asked “Thinking about these symptoms [of anxiety or depression], in what ways have these symptoms interfered with your life (e.g., day-today activities, work, social activities)?”. Interviewers were trained to thoroughly examine impairment from reported symptoms *specific to that disorder* and clearly separate it from distress or symptom severity using follow-up probing. Inter-rater reliability was excellent for all items (ICCs: GAD = 0.89, MDD = 0.93, PDD = 0.96).

##### 2.3.2.2. World Health Organisation Disability Assessment Schedule 2.0 (WHODAS) (Üstun and Kennedy, 2009).

The WHODAS is a 36-item measure of global functioning and disability, assessing six different domains: Cognition, Mobility, Self-care, Getting Along, Life Activities and Participation. The original version was used. At baseline, participants rated difficulty due to health conditions (including mental and physical conditions) over the past month, using a 5-point Likert scale from 1 (None) to 5 (Extreme or cannot do). Ratings were summed for a total score, with a higher score reflecting greater impairment. Internal consistency was excellent (Cronbach’s α = 0.95), in line with previous studies of adults aged 19 to 103 (*N* = 3482) (Noŕen et al., 2023).

#### 2.3.3. Wellbeing

##### 2.3.3.1. Mental Health Continuum-Short Form (MHC-SF) (Lamers et al., 2011).

The MHC-SF consists of 14 items that assess experiences over the past month, using a 6-point Likert scale from 0 (Never) to 5 (Everyday), including emotional, psychological, and social wellbeing. The original version was used. This measure was administered at the baseline and follow-up survey (1 month later), and we analysed the subscale scores. Internal consistency at each timepoint was excellent (Cronbach’s α = 0.93–0.94), similar to previous studies conducted with large samples of Dutch adults (*N* = 1662) (Lamers et al., 2011).

#### 2.4. Data analysis

Mplus version 8.4 (Muthén & Muthén, 2012–2019) was used to perform all analyses. Confirmatory factor analysis (CFA) was first conducted to examine latent variables for interview-rated and self-reported symptoms, self-reported wellbeing, and self-reported impairment. In the primary models, the anxiety factor was indicated by ADIS-5 GAD subscale scores (worry uncontrollability, excessiveness, and physical symptoms), and the depression factor was indicated by ADIS-5 MDD and PDD symptom sum scores. In the secondary models, the symptom factors were indicated by IDAS-II Anxious Mood items and Dysphoria items, while Impairment was indicated by the six WHODAS 2.0 subscale scores. In all models, Wellbeing was indicated by subscale scores from MHC-SF (Emotional, Psychological and Social Wellbeing). Robust maximum likelihood estimation (MLR) was used, and good model fit was defined as a root mean square error of approximation (RMSEA) around 0.100 or smaller, comparative fit index (CFI) greater than 0.90–0.95, and standardised root mean square error residual (SRMR) values close to 0.08 or below (Brown, 2015). Residual correlations were consulted to determine local fit, where correlations <0.100 indicates good local fit (Kline, 2024) (see online supplement). R^2^ was also consulted to index the variance accounted for by each model, with 0.14 considered small, 0.39 medium, and 0.59 large (Cohen, 1988; Fairchild et al., 2009).

Structural equation modelling that incorporated mediation was conducted using full information Bayesian estimation, which accounts for missing data and can handle non-normal distributions without biasing parameter estimates.^2^ To test the hypothesis that the relationship between symptoms and wellbeing is mediated by impairment, the primary model consisted of latent interview-rated anxiety and depression as the independent variables, observed interview-rated impairment as the mediator, and latent self-reported wellbeing as the dependent variable. The secondary models consisted of latent self-reported symptoms as the independent variables, latent self-reported impairment as the mediator, and latent self-reported wellbeing as the dependent variable.^3^ The secondary models controlled for the effect of self-reported physical health conditions, given that the WHODAS asks about impairment from physical health. The Mplus syntax for the main models is provided in Supplementary Table 3. Exploratory analyses were run with each concurrent model examining separate wellbeing facets (latent emotional, social, psychological).^4^ To examine longitudinal persistence of associations, models were tested with wellbeing assessed concurrently (all variables at baseline) and prospectively (wellbeing at follow-up), with and without controlling for baseline wellbeing. Last, three pre-registered exploratory analyses were conducted to assess impacts of individual interviewer-rated symptoms in mediation models for each disorder (symptoms and impairment items modelled as observed, wellbeing modelled as latent).

## 3. Results

### 3.1. Descriptive statistics

Table 1 displays descriptive statistics and the correlation matrix between variables. The associations between all symptom scores and wellbeing subscales were negative and ranged from small to moderate in size (*r*s = −0.27 to −0.59). There were moderate to large positive associations of symptom measures and impairment measures (*r*s = 0.48 to 0.86), and small to moderate negative associations of impairment measures and wellbeing subscales (*r*s = −0.30 to −0.50). The supplementary file (Table 1) shows means, standard deviations, and ranges for ADIS-5 item-level symptom ratings included in exploratory analyses. Most symptoms encompassed the full range of severity, but means were typically low (i.e., 1–2).

### 3.2. Primary models: interviewer-rated

Primary models were run as pre-registered, with latent interview-rated anxiety and depression predicting wellbeing via impairment. However, interpretation of results from this model was not tenable because the depression factor and depression impairment score—where factors represent the shared variance for MDD and PDD— were almost perfectly correlated (*r* = 0.97), so the model would not converge on a proper solution. Alternative models were run (e.g., latent Distress factor, separate models with latent MDD and PDD) but correlations between factors remained high (*r*s = 0.90 to 0.95). Correlations between individual symptom composites (i.e., separating MDD and PDD) and associated impairment rating were somewhat weaker, suggesting multicollinearity was less of a concern, so separate models were run for each disorder to facilitate interpretable effects. Additionally, for the GAD model, modification indices suggested correlating the worry excessiveness and uncontrollability composites, which improved fit and was theoretically justified given that both composites are functions of worry severity (Rutter and Brown, 2015). The CFA demonstrated excellent fit (Table 2) and standardised factor loadings were strong and significant (> 0.80).

Table 2 presents concurrent primary mediation models for interviewer-rated GAD, MDD, and PDD symptoms. Fit was good for each model, and between 21.8%–30.0% of variance in Wellbeing was explained by each model. Significant direct and total effects in expected directions were observed for each model. Partial mediation was observed in the GAD and MDD models, but not the PDD model. Full mediation was also observed in the MDD prospective model when baseline wellbeing was uncontrolled, but not when controlling for baseline wellbeing. The indirect effect for GAD, however, did not persist in prospective models (see Table 3 for all prospective models). At the facet level, impairment significantly mediated the relationship between anxiety and social wellbeing, as well as MDD symptoms and all facets of wellbeing (see Supplementary Table 2).

### 3.3. Secondary models: self-report

Secondary models were run with self-reported Anxiety and Depression modelled separately, to be consistent with the primary models. The results of concurrent models are also presented in Table 2, showing acceptable fit.

The direct, total, and indirect effect were significant in the Anxiety model, consistent with partial mediation as observed in concurrent primary model. However, the indirect effect was not significant in the Depression model. Thirty-five percent of variance in wellbeing was explained in the Anxiety model, and 40.6% in the Depression model. Indirect effects were present in the Anxiety prospective model without baseline wellbeing, but not in any Depression prospective models nor the Anxiety prospective model when controlling for baseline wellbeing (see Table 3). At the facet level, impairment significantly mediated the relationship between anxiety and all facets of wellbeing (see Supplementary Table 2).

### 3.4. Exploratory analyses

Table 4 presents results from pre-registered exploratory analyses examining the contribution of individual interviewer-rated symptoms. In the GAD model, uncontrollable worry and most physical symptoms (all except muscle tension) significantly predicted lower wellbeing. However, excessiveness of worry did not significantly predict impairment or wellbeing. Significant indirect pathways via impairment were present for uncontrollability, restlessness, irritability, sleep disturbance, fatigue, and trouble concentrating due to worry. In the MDD model, the following symptoms significantly predicted impairment directly, and their association with wellbeing was fully mediated by impairment: depressed mood, anhedonia, trouble concentrating, feelings of guilt/blame, and fatigue. In the PDD model, sleep disturbance, trouble concentrating, and pessimism/hopelessness had significant direct effects on wellbeing, but no significant indirect effects were observed.

## 4. Discussion

The present study broadens our understanding of how and why symptoms influence wellbeing, demonstrating the important role of impairment. Findings suggest that an impaired ability to function in key life domains is associated with greater major depressive symptoms and generalised anxiety, which contributes to a lower sense of wellbeing. This highlights the need to consider not only the impact of symptom severity on wellbeing, but also the extent to which symptoms, particularly anxiety, impact one’s life and the downstream detrimental effects of this on wellbeing.

### 4.1. Depression symptoms

Impairment mediated the association between MDD symptoms and lower wellbeing (more specifically, core symptoms of depressed mood and anhedonia, trouble concentrating, and fatigue). However, more general dysphoria symptoms (self-reported) had direct effects on well-being, but this was not mediated by self-reported impairment. Although the MDD module and IDAS-II Dysphoria scale are similar in content and timeframe assessed, the self-reported Dysphoria scale lacks assessment of some symptoms that drive impairment, such as fatigue.

Nevertheless, specific MDD symptoms appear to drive the effects on wellbeing in part because of their relatively strong impact on impairment, which is consistent with prior findings (Tanner et al., 2019; Fried and Nesse, 2014). A novel finding was that feelings of self-blame and guilt also indirectly impacted wellbeing via impairment, perhaps related to increased tendencies to withdraw when experiencing these feelings (Duan et al., 2022). Our findings for MDD are consistent with prior results that impairment mediated the association between individual MDD symptoms and lower quality of life (Zhou et al., 2022). This suggests that impairment contributes to the association of major depression symptoms with wellbeing and related constructs, in both clinical and non-clinical samples.

Although PDD symptoms overall and individually had direct effects on wellbeing, their association was not mediated by levels of impairment. Though counter to our hypotheses, this may be due to the chronicity of symptoms in PDD, which by definition must be present most days for at least two years. In the context of long-term symptoms, it may be difficult to identify impairment relative to one’s ‘norm’, which is often person- and context-dependent. Moreover, because PDD is assessed over a much larger timeframe than MDD and the IDAS (2 years vs. 2 weeks) and wellbeing (past month), acute declines in functioning and subsequent wellbeing might be more apparent in shorter timeframes of assessment. In addition, the assessment of impairment focused on current and recent experiences, meaning that more distal but severe impairment was likely missed or under-represented in these ratings. Thus, it is possible that PDD symptoms impact wellbeing via changes in functioning over time, but more granular temporal assessments of symptoms may be needed to capture these fluctuations than provided in the current study.

### 4.2. Generalised anxiety symptoms

Across concurrent models, anxiety symptoms and associated impairment explained moderate variance in wellbeing, and impairment partially mediated the relationship between symptoms and wellbeing. This was driven by difficulty controlling worries, and associated restlessness, irritability, sleep disturbance, fatigue, and difficulty concentrating due to worry. This partially aligns with previous studies regarding impairment from anxiety-related restlessness and concentration symptoms (Romera et al., 2011; Tanner et al., 2019), and these findings provide insight into the specific aspects of anxiety that affect wellbeing through impairment.

Notably, anxiety symptoms were strongly related to impairment in the present study, with comparable effect sizes to depression, revealing a stronger relationship than previously reported for generalised anxiety (McKnight et al., 2016, *r* = 0.31). Moreover, the relationship between anxiety and impairment is complex, as it is possible for people to perceive their anxiety as helpful and therefore not impairing (McKnight et al., 2016). Future research should consider this nuance and explore impairment in other anxiety disorders, as this is less understood.

### 4.3. Prospective effects

The effects for prospective models presented a mixed picture, based on whether baseline wellbeing was controlled for and the measures used. When baseline wellbeing was not accounted for, the mediating effect of impairment prospectively predicted wellbeing several weeks later. Specifically, impairment partially mediated the relationship between MDD symptoms (interviewer-rated only) and GAD symptoms (self-reported only) and later levels of wellbeing, but not PDD symptoms (consistent with the cross-sectional PDD models). The absence of a significant result in the GAD primary model may be due to the different time periods and content assessed. That is, general anxious mood/tendencies may be briefer and reflect elevations in symptoms (captured by IDAS Anxious Mood), compared to GAD-specific worry domains and symptoms over the past six months (captured by ADIS-5).

However, across all models, mediation did not persist when controlling for baseline wellbeing. As such, it is possible that the prospective effect observed previously primarily reflects the stability of wellbeing over time. Along these lines, it is important to note that wellbeing was highly stable over time in the present study (*r* = 0.72–0.80), and so there was little change to predict. As such, it is unclear from our results whether a) symptoms do not lead to later change in wellbeing via impairment, or b) such prospective indirect effects may exist but could not be detected in these data given a restriction of range in wellbeing change. Further research is needed to assess the longitudinal dynamics of these constructs, perhaps over longer time periods when greater change in wellbeing can be expected or in contexts that are associated with fluctuations in wellbeing (e.g., before and during a stressful period).

### 4.4. Clinical implications

Improved functioning and wellbeing are regarded as important treatment goals (Sheehan et al., 2017; Kettler, 2021), but it remains unclear how to enhance or maintain wellbeing for individuals with persistent or worsening symptoms. Our findings highlight the role of functioning, given that impairment tends to be higher in clinical samples and persists after symptom recovery (Hirschfeld et al., 2002). It may be difficult to improve wellbeing if impairment is high and not assessed or addressed adequately, even when symptoms improve. Moreover, it may be beneficial to tailor treatment approaches by focusing on reducing individual symptoms that contribute most strongly to functional difficulties in the patients’ daily lives. Assessing impairment, in addition to symptom levels, initially and throughout therapy would allow clinicians to monitor progress in this domain and adjust the approach accordingly where needed to enhance functioning.

It is important to note the wide variation in impairment levels in the broader literature, particularly in subclinical and non-clinical populations. Although symptoms and impairment were highly correlated in the present study, symptoms are not *always* closely linked to impairment, and many individuals report symptoms with very little to no negative impairment and can still receive a diagnosis (e.g., 25% of patients with MDD reported no impairment; Zimmerman et al., 2006). This provides an avenue for prevention and treatment interventions whereby moderating factors could be targeted to buffer against symptoms impacting functioning and thus prevent or reduce detriments to well-being, ultimately building resilience and positive adaptation to symptoms. Further research is needed to determine the contexts and characteristics of people where symptoms do not impair functioning and investigate whether symptoms have reduced influence on wellbeing in such circumstances.

### 4.5. Limitations and future directions

Numerous limitations should be considered. Starting with measurement issues, the correlation between the interview-assessed symptoms and impairment likely was very strong in part because impairment for each disorder was assessed immediately following assessment of symptoms in the interview (rather than across a large host of randomly-ordered self-report measures with different response formats). High symptom-impairment correlations in the interview data (0.83–0.86) mean the primary model mediation results could partly reflect measurement confounding, rather than a distinct mechanistic pathway. Secondary self-report models, where impairment was measured with a separate instrument (WHODAS), provide a cleaner test, and anxiety effects were corroborated. Thus, results of the primary depression models should be interpreted with caution. Impairment was also assessed somewhat differently in the interview and self-report, with the WHODAS capturing a wide range of sources of impairment and therefore less correlated with symptoms. More broadly, a minority of items were similar across measures in this study, including ADIS-5 modules. Even though interviewers were trained to disentangle symptoms of anxiety vs. depression, some overlap may have been present.

The findings were consistent across primary and secondary models for anxiety, but more mixed for depression, suggesting that depression effects might be small and/or depend on the population or design of the study, and thus should be replicated in future studies. In addition, the sample largely consisted of White and Asian participants residing in Australia, so generalizability to other cultures and settings is unknown. In particular, research in countries where impairment may be defined or viewed differently due to cultural norms and values would be important. Future studies should also examine these models in clinical samples with varied disorders (including other anxiety disorders) to reduce floor effects in symptom severity and impairment and test generalizability at high symptom severity. Finally, as mentioned previously, the current study assessed wellbeing after one month, but future studies might examine effects at smaller timescales (e.g., days, weeks) or longer term (e.g., months, years) to identify more fine-grained details or bigger picture trends, as well as potential bidirectional associations or feedback loops (Grant et al., 2013; Lamers et al., 2015).

## 5. Conclusion

Using a variety of measures and statistical models, this study revealed that impairment plays a consistent role in the association between generalised anxiety symptoms with lowered wellbeing, as well as a potential smaller role for depressive symptoms and prospective effects. These findings suggest that impairment from symptoms is an important factor connecting symptoms to reduced wellbeing particularly at the same time point, and it should be considered when trying to understand and promote wellbeing in the context of symptoms.

## Supplementary Material

1

## Figures and Tables

**Table 1:** Descriptive statistics for variables in the main analyses.

Variable	*M*	*SD*	Min.	Max.	1	2	3	4	5	6	7	8	9	10	11	12	13	14	15	16	17
1. GAD Excessiveness (ADIS-5)	11.74	7.57	0	47	–																
2. GAD Uncontrollability (ADIS-5)	7.89	7.21	0	42	0.89	–															
3. GAD Physical symptoms (ADIS-5)	10.45	8.10	0	42	0.75	0.76	–														
4. MDD sum (ADIS-5)	10.52	11.12	0	54	0.60	0.61	0.68	–													
5. PDD sum (ADIS-5)	11.80	10.39	0	50	0.62	0.65	0.70	0.72	–												
6. GAD Impairment (ADIS-5)	1.92	1.77	0	8	0.70	0.71	0.79	0.65	0.67	–											
7. MDD Impairment (ADIS-5)	1.17	1.64	0	8	0.48	0.50	0.54	0.83	0.64	0.60	–										
8. PDD Impairment (ADIS-5)	1.64	1.81	0	7	0.54	0.56	0.59	0.68	0.86	0.62	0.66	–									
9. IDAS Dysphoria	21.08	7.92	9	41	0.60	0.62	0.62	0.64	0.64	0.63	0.61	0.58	–								
10. IDAS Anxious Mood	18.50	7.03	7	35	0.64	0.64	0.63	0.57	0.58	0.60	0.52	0.48	0.84	–							
11. WHODAS sum	64.58	20.87	36	138	0.57	0.60	0.59	0.61	0.64	0.60	0.58	0.57	0.74	0.63	–						
12. TO Emotional Wellbeing (MHC-SF)	8.98	3.57	0	15	−0.33	−0.42	−0.41	−0.46	−0.52	−0.43	−0.47	−0.42	−0.59	−0.50	−0.48	–					
13. TO Social Wellbeing (MHC-SF)	11.04	5.86	0	25	−0.27	−0.34	−0.34	−0.37	−0.45	−0.36	−0.36	−0.34	−0.45	−0.39	−0.41	0.72	–				
14. TO Psychological Wellbeing (MHC-SF)	17.13	6.61	2	30	−0.32	−0.39	−0.36	−0.41	−0.50	−0.39	−0.40	−0.39	−0.55	−0.46	−0.49	0.81	0.77	–			
15. T1 Emotional Wellbeing (MHC-SF)	8.97	3.39	0	15	−0.33	−0.37	−0.36	−0.38	−0.47	−0.39	−0.42	−0.39	−0.56	−0.50	−0.50	0.72	0.60	0.67	–		
16. T1 Social Wellbeing (MHC-SF)	11.01	5.68	0	25	−0.27	−0.32	−0.32	−0.31	−0.39	−0.32	−0.32	−0.30	−0.44	−0.40	−0.42	0.62	0.80	0.67	0.73	–	
17. T1 Psychological Wellbeing (MHC-SF)	16.76	6.35	2	30	−0.29	−0.35	−0.34	−0.34	−0.44	−0.35	−0.35	−0.33	−0.51	−0.45	−0.46	0.63	0.66	0.75	0.81	0.83	–

Note. All correlations were significant at *p* <.001.

**Table 2:** Main analyses concurrent models: Model fit statistics and regression coefficients for each model.

Model	Model fit									Estimates					
		N	FP	dfM	RMSEA	CFI	SRMR	PPP	R^2^		*a* Path (symptoms ➔ impairment)	*b* Path (impairment ➔ wellbeing)	*c’* Path (symptoms ➔ wellbeing)	*c* Total effect	*ab* Indirect effect
Primary models															
GAD	Measurement	408	19	8	0.068	0.990	0.030	–	–						
	Structural	408	24	–	0.035	0.997	–	0.154	0.220	Standardised:	0.861 (0.019) [0.817, 0.894]	−0.221 (0.071) [−0.364, −0.092]	−0.264 (0.082) [0.421, −0.092]	−0.454 (0.045) [−0.529, −0.358]	−0.190 (0.062) [−0.314, −0.079]
										Unstandardised:	0.204 (0.009) [0.186, 0.225]	−0.393 (0.122) [−0.625, −0.156]	−0.111 (0.035) [-/178, −0.035]	−0.190 (0.021) [−0.229, −0.148]	−0.080 (0.025) [0.128, −0.034]
MDD	Measurement	370	9	0	0.000	1.00	0.000	–	–						
	Structural	407	14	–	0.067	0.995	–	0.125	0.218	Standardised:	0.832 (0.013) [0.803, 0.854]	−0.260 (0.089) [−0.418, −0.073]	−0.228 (0.089) [−0.416, −0.042]	−0.442 (0.044) [−0.519, −0.346]	−0.216 (0.075) [−0.348, −0.060]
										Unstandardised:	0.123 (0.004) [0.115, 0.132]	−0.490 (0.174) [−0.826, −0.130]	−0.064 (0.025) [−0.117, −0.012]	−0.125 (0.015) [−0.155, −0.094]	−0.060 (0.022) [−0.102, −0.016]
PDD	Measurement	370	9	0	0.000	1.00	0.000	–	–						
	Structural	407	14	–	0.000	1.00	–	0.500	0.300	Standardised:	0.858 (0.010) [0.837, 0.877]	0.145 (0.091) [−0.031, 0.323]	−0.664 (0.085) [−0.827, −0.439]	−0.542 (0.037) [−0.606, −0.462]	0.124 (0.078) [−0.027, 0.276]
										Unstandardised:	0.150 (0.004) [0.142, 0.159]	0.249 (0.157) [−0.052, 0.559]	−0.199 (0.028) [−0.255, −0.142]	−0.162 (0.015) [−0.190, −0.131]	0.037 (0.024) [−0.008, 0.084]
Secondary models															
Anxiety	Measurement	408	51	101	0.061	0.959	0.043	–	–						
	Structural	322	52	–	0.063	0.961	–	0.000	0.345	Standardised:	0.682 (0.033) [0.609, 0.743]	−0.401 (0.073) [−0.534, −0.252]	−0.224 (0.074) [−0.365, −0.081]	−0.495 (0.047) [0.584, −0.403]	−0.273 (0.052) [−0.371, −0.169]
										Unstandardised:	2.915 (0.274) [2.417, 3.473]	−0.377 (0.074) [−0.518, −0.238]	−0.894 (0.313) [−1.541, −0.327]	−1.999 (0.260) [−2.556, −1.533]	−1.088 (0.231) [−1.563, −0.0680]
Depression	Measurement	408	57	132	0.072	0.924	0.050	–	–						
	Structural	322	58	–	0.075	0.929	–	0.000	0.406	Standardised:	0.827 (0.026) [0.767, 0.870]	−0.113 (0.114) [0.310, 0.123]	−0.532 (0.106) [−0.730, −0.331]	−0.623 (0.041) [−0.700, −0.538]	−0.094 (0.094) [−0.225, 0.110]
										Unstandardised:	4.408 (0.503) [3.505, 5.386]	−0.108 (0.110) [−0.306, 0.116]	−2.688 (0.622) [−3.873, −1.577]	−3.141 (0.415) [−4.046, −2.408	−0.473 (0.479) [−1.346, 0.503]

Note. N = number of observations, DfM = model degrees of freedom, FP = Number of free parameters. For Bayesian estimation, Degrees of Freedom are not reported because the chi-square test that includes degrees of freedom are not used. Round brackets = Posterior S.D. Square brackets = 95% credibility intervals. Bolded = statistically significant, *p* < .05.

**Table 3:** Prospective models with wellbeing assessed 1 month later: Model fit statistics and standardised regression coefficients for each model.

Model	Model fit									Estimates					
		N	FP	dfM	RMSEA	CFI	SRMR	PPP	R^2^		*a* Path (symptoms ➔ impairment)	*b* Path (impairment ➔ wellbeing)	*c’* Path (symptoms ➔ wellbeing)	*c* Total effect	*ab* Indirect effect
Primary models															
GAD *WB controlled*	Measurement	408	34	20	0.032	0.997	0.022	–	–						
	Structural	408	38	–	0.000	1.00	–	0.312	0.682	Standardised:	0.866 (0.017) [0.831, 0.896]	−0.032 (0.077) [−0.162, 0.136]	−0.010 (0.085) [−0.186, 0.145]	−0.038 (0.043) [−0.117, 0.050]	−0.028 (0.067) [−0.142, 0.119]
										Unstandardised:	0.207 (0.009) [0.190, 0.225]	−0.052 (0.126) [−0.264, 0.224]	−0.004 (0.033) [−0.073, 0.057]	−0.015 (0.017) [−0.046, 0.019]	−0.011 (0.026) [−0.056, 0.047]
*WB not controlled*	Measurement	407	20	7	0.038	0.998	0.021	–	–						
	Structural	407	24	–	0.000	1.00	–	0.388	0.182	Standardised:	0.861 (0.018) [0.823, 0.893]	−0.081 (0.139) [−0.393, 0.154]	−0.342 (0.139) [−0.599, −0.040]	−0.417 (0.052) [−0.516, −0.309]	−0.070 (0.119) [−0.334, 0.132]
										Unstandardised:	0.205 (0.009) [0.187, 0.224]	−0.132 (0.226) [−0.642, 0.257]	−0.131 (0.055) [−0.238, −0.016]	−0.161 (0.023) [−0.205, −0.114]	−0.027 (0.046) [−0.128, 0.053]
MDD *WB controlled*	Measurement	392	22	5	0.000	1.00	0.010	–	–						
	Structural	407	27	–	0.119	0.962	–	0.000	0.671	Standardised:	0.831 (0.012) [0.807, 0.854]	−0.076 (0.067) [−0.197, −0.057]	0.039 (0.049) [−0.066, 0.127]	−0.007 (0.027) [−0.061, 0.047]	−0.045 (0.040) [−0.117, 0.033]
										Unstandardised:	0.124 (0.004) [0.115, 0.132]	−0.132 (0.117) [−0.349, 0.102]	0.014 (0.018) [−0.024, 0.047]	−0.003 (0.010) [−0.021, 0.017]	−0.016 (0.014) [−0.043, 0.012]
*WB not controlled*	Measurement	330	9	0	0.000	1.00	0.000	–	–						
	Structural	407	14	–	0.058	0.994	–	0.000	0.137	Standardised:	0.832 (0.011) [0.813, 0.854]	−0.282 (0.074) [−0.419, −0.091]	−0.088 (0.074) [−0.236, 0.027]	−0.330 (0.043) [−0.411, −0.243]	−0.233 (0.062) [−0.345, −0.076]
										Unstandardised:	0.123 (0.004) [0.116, 0.133]	−0.490 (0.125) [−0.717, −0.165]	−0.023 (0.019) [−0.062, 0.007]	−0.084 (0.011) [−0.108, 0.061]	−0.060 (0.016) [−0.090, −0.020]
PDD *WB controlled*	Measurement	392	22	5	0.000	1.00	0.010	–	–						
	Structural	407	27	–	0.137	0.953	–	0.000	0.656	Standardised:	0.858 (0.011) [0.835, 0.877]	0.065 (0.071) [−0.071, 0.204]	−0.093 (0.054) [−0.203, 0.012]	−0.053 (0.031) [−0.113, 0.010]	0.040 (0.043) [−0.043, 0.125]
										Unstandardised:	0.150 (0.005) [0.141, 0.159]	0.100 (0.111) [−0.104, 0.332]	−0.036 (0.020) [−0.076 0.005]	−0.020 (0.012) [−0.041, 0.004]	0.015 (0.017) [−0.016, 0.049]
*WB not controlled*	Measurement	330	9	0	0.000	1.00	0.000	–	–						
	Structural	407	14	–	0.051	0.997	–	0.135	0.266	Standardised:	0.858 (0.011) [0.837, 0.877]	0.191 (0.104) [−0.017, 0.396]	−0.673 (0.090) [−0.836, −0.485]	−0.503 (0.036) [−0.575, −0.438]	0.163 (0.089) [−0.015, 0.336]
										Unstandardised:	0.150 (0.004) [0.141, 0.158]	0.308 (0.169) [−0.030, 0.631]	−0.189 (0.029) [−0.246, −0.135]	−0.141 (0.014) [−0.174, −0.119]	0.046 (0.025) [−0.004, 0.096]
Secondary models															
Anxiety	Measurement	408	63	146	0.074	0.935	0.046	–	–						
*WB controlled*	Structural	322	63	–	0.076	0.936	–	0.000	0.709	Standardised:	0.691 (0.033) [0.617, 0.751]	−0.079 (0.062) [−0.205, 0.038]	−0.026 (0.056) [−0.139, 0.086]	−0.082 (0.046) [−0.174, 0.011]	−0.307 (,176) [−0.656, 0.040]
										Unstandardised:	3.053 (0.299) [2.546, 3.673]	−0.098 (0.216) [−0.530, 0.341]	−0.098 (0.216) [−0.530, 0.341]	−0.307 (0.176) [−0.656, 0.040]	−0.201 (0.165) [−0.564, 0.097]
*WB not controlled*	Measurement	408	51	101	0.062	0.959	0.046	–	–						
	Structural	322	52	–	0.056	0.969	–	0.000	0.314	Standardised:	0.680 (0.035) [0.603, 0.743]	−0.392 (0.076) [−0.535, −0.245]	−0.201 (0.083) [−0.367, −0.054]	−468 (0.053) [−0.569, −0.356]	−0.265 (0.054) [−0.372, −0.160]
										Unstandardised:	2.835 (0.249) [2.398, 3.372]	−0.345 (0.069) [−0.482, −0.211]	−0.727 (0.330) [−1.410, −0.185]	−1.707 (0.287) [−2.397, −1.247]	−0.974 (0.215) [−1.434, −0.563]
Depression	Measurement	408	69	183	0.076	0.910	0.052	–	–						
*WB controlled*	Structural	322	69	–	0.078	0.917	–	0.000	0.708	Standardised:	0.832 (0.023) [0.784, 0.872]	−0.066 (0.083) [−0.236, 0.095]	−0.015 (0.096) [−0.198, 0.187]	−0.066 (0.059) [−0.184, 0.045]	−0.054 (0.069) [−0.201, 0.075]
										Unstandardised:	4.451 (0.408) [3.708, 5.265]	−0.056 (0.072) [−0.202, 0.080]	−0.068 (0.445) [−0.893, 0.899]	−0.301 (0.271) [−0.856, 0.214]	−0.246 (0.321) [−0.899, 0.349]
*WB not controlled*	Measurement	408	57	132	0.070	0.929	0.050	–	–						
	Structural	322	58	–	0.068	0.939	–	0.000	0.358	Standardised:	0.830 (0.023) [0.777, 0.865]	−0.194 (0.113) [−0.406, 0.031]	−0.416 (0.111) [−0.623, −0.192]	−0.574 (0.042) [−0.653, −0.486]	−0.161 (0.094) [−0.338, 0.025]
										Unstandardised:	4.772 (0.394) [4.038, 5.551]	−0.170 (0.100) [−0.352, 0.027]	−2.036 (0.608) [−3.260, −0.939]	−2.880 (0.408) [−3.707, −2.148]	−0.801 (0.047) [−1.727, 0.132]

Note. N = number of observations, DfM = model degrees of freedom, FP = Number of free parameters. WB = Wellbeing at T0 (baseline).

For Bayesian estimation, Degrees of Freedom are not reported because the chi-square test that includes degrees of freedom are not used.

Round brackets = Posterior S.D. Square brackets = 95% credibility intervals.

Bolded = statistically significant, p < .05.

**Table 4:** Symptom-level Analyses: Model fit statistics and standardised regression coefficients for each model.

Variable	RMSEA	CFI	PPP	R^2^	*a* path, estimate [95% CI] (each symptom ➔ impairment)	*b* path, estimate [95% CI] (impairment ➔ wellbeing)	*c*’ Direct effect, estimate [95% CI] (each symptom ➔ wellbeing)	*c* Total effect, estimate [95% CI]	*ab* Indirect effect, estimate [95% CI]
GAD Model	0.033	0.994	0.096	**0.261**		**−0.191 [0.359, −0.031]**			
Uncontrollable worrying					**0.157 [0.035, 0.273]**		**−0.362 [−0.532, −0.157]**	**−0.396 [−0.562, −0.182]**	**−0.027 [−0.070, −0.003]**
Excessive worrying					0.110 [−0.007, 0.224]		0.163 [−0.009, 0.354]	0.142 [−0.035, 0.334]	−0.019 [−0.059, 0.003]
Restlessness					**0.178 [0.104, 0.239]**		−0.078 [−0.197, 0.047]	−0.108–0.229, 0.012]	**−0.032 [−0.068, −0.003]**
Irritability					**0.100 [0.043, 0.159]**		−0.025 [−0.124, 0.081]	−0.042 [−0.143, 0.058]	**−0.018 [−0.042, −0.002]**
Sleep disturbance					**0.080 [0.016, 0.138]**		−0.091–0.182, 0.026]	−0.105 [−0.192, 0.012]	**−0.014 [−0.040, −0.001]**
Fatigue					**0.252 [0.179, 0.317]**		0.033 [−0.096, 0.161]	−0.017 [−0.134, 0.101]	**−0.047 [−0.097, −008]**
Concentration trouble					**0.235 [0.163,0.303]**		−0.093 [−0.219, 0.028]	**−0.139 [−0.256, −0.017]**	**−0.045 [−0.090, −0.006]**
Muscle tension					−0.038 [−0.101, 0.022]		0.105 [−0.007, 0.208]	0.114 [0.000, 0.220]	0.006 [−0.004, 0.025]
MDD Model	0.020	0.998	0.250	**0.259**		**−0.202**			
Depressed mood					**0.370 [0.2890, 0.443]**	**[−0.378, −0.027]**	−0.067 [−0.231, 0.089]	−0.146 [−0.295, 0.010]	**−0.077 [−0.140, −0.011]**
Anhedonia					**0.313 [0.244, 0.374]**		−0.091 [−0.223, 0.057]	**−0.153 [−0.268, −0.034]**	**−0.063 [−0.120, −0.008]**
Sleep disturbance					0.050 [−0.006, 0.105]		−0.058 [163, 0.046]0	−0.071 [−0.171, 0.036]	−0.009 [−0.030, 0.001]
Weight change					0.035–0.021, 0.097]		.092 [−0.044, 0.203]	0.085 [−0.050, 0.197]	−0.006 [−0.026, 0.004]
Psychomotor change					0.029 [−0.027, 0.090]		−0.095 [−0.224, 0.016]	−0.102 [−0.231, 0.011]	−0.005 [0.025, 0.006]
Concentration trouble					**0.102 [0.036, 0.167]**		−0.074–0.198, 0.051]	−0.096 [−0.223, 0.027]	**−0.200 [−0.046, −0.001]**
Suicidality					−0.023 [−0.081, 0.033]		−0.066 [−0.166, 0.038]	−0.062 [−0.164, 0.044]	−0.004 [−0.007, 0.020]
Guilt/blame					**0.099 [0.039, 0.159]**		−0.014 [−0.119, 0.113]	−0.034 [−0.138, 0.081]	**−0.019 [−0.049, −0.002]**
Fatigue					**0.115 [0.042, 0.184]**		−0.026 [−0.157, 0.115]	−0.049 [−0.182, 0.088]	**−0.022 [−0.051, −0.002]**
PDD Model	0.015	1.00	0.364	**0.304**		0.173 [−0.020, 0.361]			
Depressed mood					**0.497 [0.415, 0.560]**		−0.144 [−0.331, 0.054]	−0.053 [−0.199, 0.100]	0.086 [−0.010, 0.188]
Appetite changes					**0.100 [0.028, 0.152]**		−0.026 [−0.134, 0.069]	−0.009 [−0.112, 0.098]	0.015 [−0.002, 0.043]
Sleep disturbance					0.044 [−0.001, 0.090]		**−0.154 [−0.286, −0.033]**	**−0.149 [−0.271, −0.026]**	0.006 [−0.002, 0.020]
Fatigue					**0.113 [0.051, 0.163]**		−0.123 [−0.249, 0.002]	−0.105 [−0.230, 0.026]	0.019 [−0.002, 0.053]
Concentration trouble					**0.077 [0.015, 0.135]**		**−0.182 [−0.367, −0.053]**	**−0.171–0.311, −0.035]**	0.011 [−0.002, 0.038]
Low self esteem					**0.100 [0.016, 0.177]**		−0.017–0.158, 0.113]	0.001 [−0.129, 0.122]	0.027 [−0.002, 0.047]
Pessimism/hopelessness					**0.139 [0.060, 0.215]**		**−0.226 [0.060, 0.215]**	**−0.196 [−0.331, −0.029]**	−0.023–0.003, 0.061]

Note. Bolded = statistically significant, *p* < .05. See Supplementary Table 4 for results of the pre-registered GAD model (physical symptom composite).

## Data Availability

Data used in the preparation of this manuscript are available to access from the National Institute of Mental Health (NIMH) Data Archive (NDA). NDA is a collaborative informatics system created by the National Institutes of Health to provide a national resource to support and accelerate research in mental health. Dataset identifier(s): 10.15154/4kb5-fw77. This manuscript reflects the views of the authors and may not reflect the opinions or views of the NIH or of the Submitters submitting original data to NDA.

## Footnotes

1 The interviewer-rated model was considered primary given the ability to probe for more detailed information and clarify responses in diagnostic interviews, which are considered ‘gold standard’ in assessing psychological symptoms (American Psychiatric Association, 2013).

2 Bayesian estimation was used for these models because it can appropriately model the asymmetric confidence interval of indirect effects, whereas this is not currently possible with MLR estimators in Mplus. As a default in Mplus, Bayesian estimation specifies non-informative priors.

3 This deviates slightly from the pre-registered secondary analyses as full structural equation modelling was requested by an anonymous reviewer, rather than the originally planned observed variables.

4 These analyses were suggested by an anonymous reviewer, and results are presented in the online supplement for interested readers.

## Appendix A. Supplementary data

Supplementary data to this article can be found online at https://doi.org/10.1016/j.jad.2026.121517.
