# Supplementary material for: Investigating functional impairment as a mediator of the association between internalising symptoms and low wellbeing
Source: J Affect Disord. Author manuscript; Available in PMC 2026 May 31. (PMC13222730; doi:10.1016/j.jad.2026.121517)
Supplement: 1 [file NIHMS2179276-supplement-1.docx]

**Supplementary Table 1**

*Means, standard deviations, and range for ADIS-5 individual symptom ratings in the exploratory analyses.*

| Symptom | *M* | *SD* | Min. | Max. |
| --- | --- | --- | --- | --- |
| **GAD Physical symptoms** |  |  |  |  |
| Restlessness | 2.05 | 2.08 | 0 | 8 |
| Irritability | 1.67 | 1.65 | 0 | 7 |
| Sleep disturbance | 2.04 | 2.1 | 0 | 8 |
| Fatigue | 1.53 | 1.87 | 0 | 8 |
| Concentration trouble | 1.89 | 1.87 | 0 | 8 |
| Muscle tension | 1.27 | 1.88 | 0 | 8 |
| **MDD Symptoms** |  |  |  |  |
| Depressed mood | 1.52 | 1.81 | 0 | 8 |
| Anhedonia | 1.11 | 1.88 | 0 | 8 |
| Weight change | 0.78 | 1.45 | 0 | 6 |
| Sleep disturbance | 1.55 | 1.84 | 0 | 7 |
| Psychomotor change | 0.84 | 1.54 | 0 | 7 |
| Fatigue | 1.38 | 1.83 | 0 | 8 |
| Guilt/blame | 1.49 | 1.9 | 0 | 7 |
| Concentration troubles | 1.24 | 1.8 | 0 | 7 |
| Suicidality | 0.26 | 0.91 | 0 | 7 |
| **PDD Symptoms** |  |  |  |  |
| Depressed mood | 1.8 | 1.82 | 0 | 7 |
| Appetite changes | 1.14 | 1.48 | 0 | 6 |
| Sleep disturbance | 1.93 | 1.93 | 0 | 8 |
| Fatigue | 1.73 | 1.93 | 0 | 8 |
| Low self esteem | 1.71 | 1.91 | 0 | 8 |
| Concentration trouble | 1.62 | 1.93 | 0 | 7 |
| Pessimism/hopelessness | 1.41 | 1.83 | 0 | 8 |

**Supplementary Table 2**

Main analyses concurrent models separated by wellbeing facet: Model fit statistics and regression coefficients for each model.

| Model | *Model fit* | | | | | | | | | *Estimates* | | | | | |
| --- | --- | --- | --- | --- | --- | --- | --- | --- | --- | --- | --- | --- | --- | --- | --- |
|  |  | N | FP | dfM | RMSEA | CFI | SRMR | PPP | R^2^ |  | *a* Path (symptoms 🡪 impairment) | *b* Path (impairment 🡪 wellbeing) | *c’* Path (symptoms 🡪 wellbeing) | *c* Total effect | *ab* Indirect effect |
| **Primary Models** | | | | | | | | | | | | | | | |
| GAD | Measurement | 408 | 58 | 112 | .064 | .946 | .045 | - | - |  |  |  |  |  |  |
|  | Structural | 408 | 64 | - | .069 | .946 | - | .000 | EWB: **.252**  SWB: **.175**  PWB: **.215** | Standardised: | **.864 (.017)**  **[.827, .895]** | EWB: -.068 (.090)  [-.223, .099]  **SWB: -.135 (.076)**  **[-.314, -.010]**  PWB: -.047 (.085)  [-.248, .079] | **EWB: -.439 (.101)**  **[-.625, -.241]**  **SWB: -.294 (.088)**  **[-.435, -.099]**  **PWB: -.419 (.090)**  **[-.564, -.212]** | **EWB: -.498 (.044)**  **[-.587, -.421]**  **SWB: -.412 (.049)**  **[-.491, -.305]**  **PWB: -.460 (.044)**  **[-.541, -.373]** | EWB: -.059 (.077)  [-.191, .086]  **SWB: -.117 (.065)**  **[-.268, -.009]**  PWB: -.040 (.073)  [-.21-, .068] |
|  |  |  |  |  |  |  |  |  |  | Unstandardised: | **.251 (.012)**  **[.224, .271]** | EWB: -.041 (.050)  [-.124, .055]  **SWB: -.091 (.050)**  **[-.203, -.006]**  PWB: -.031 (.058)  [-.171, .056] | **EWB: -.071 (.017)**  **[-.103, -.038]**  **SWB: -.057 (.019)**  **[-.091, -.018]**  **PWB: -.080 (.020)**  **[-.117, -.038]** | **EWB: -.083 (.009) [-.099, -.064]**  **SWB: -.080 (.013)**  **[-.102, -.058]**  **PWB: -.088 (.011)**  **[-.112, -.068]** | EWB: -.010 (.013)  [-.031, .014]  **SWB: -.022 (.012)**  **[-.051, -.002]**  PWB: -.007 (.014)  [-.042, .013] |
| MDD | Measurement | 408 | 45 | 74 | .071 | .935 | .043 | - | - |  |  |  |  |  |  |
|  | Structural | 407 | 54 | - | .077 | .937 | - | .000 | EWB: **.271**  SWB: **.177**  PWB: **.211** | Standardised: | **.829 (.033)**  **[.804, .853]** | **EWB: -.278 (.084)**  **[-.442, -.115]**  **SWB: -.212 (.087)**  **[-.384, -.035]**  **PWB: -.208 (.087)**  **[-.383, -.035]** | **EWB: -.265 (.083)**  **[-.419, -.100]**  **SWB: -.225 (.087)**  **[-.387, -.059]**  **PWB: -.277 (.088)**  **[-.443, -.102]** | **EWB: -.493 (.040)**  **[-.572, -.420]**  **SWB: -.402 (.045)**  **[-.482, -.312]**  **PWB: -.442 (.045)**  **[-.526, -.348]** | **EWB: -.233 (.070)**  **[-.369, -.096]**  **SWB: -.176 (.073)**  **[-.318, -.029]**  **PWB: -.171 (.073)**  **[-.315, -.029]** |
|  |  |  |  |  |  |  |  |  |  | Unstandardised: | **.123 (.004)**  **[.115, .131]** | **EWB: -.165 (.051)**  **[-.265, -.069]**  **SWB: -.155 (.064)**  **[-.280, -.026]**  **PWB: -.147 (.063)**  **[-.275, -.025]** | **EWB: -.023 (.008)**  **[-.038, -.009]**  **SWB: -.024 (.010)**  **[-.043, -.006]**  **PWB: -.029 (.010)**  **[-.049, -.011]** | **EWB: -.043 (.005)**  **[-.054, -.035]**  **SWB: -.043 (.006)**  **[-.054, -.032]**  **PWB: -.047 (.006)**  **[-.059, -.037]** | **EWB: -.020 (.006)**  **[-.033, -.008]**  **SWB: -.019 (.008)**  **[-.035, -.003]**  **PWB: -.018 (.008)**  **[-.034, -.003]** |
| PDD | Measurement | 408 | 45 | 74 | .071 | .935 | .043 | - | - |  |  |  |  |  |  |
|  | Structural | 407 | 54 | - | .079 | .934 | - | .000 | EWB: **.327**  SWB: **.241**  PWB: **.304** | Standardised: | **.857 (.010)**  **[.837, .879]** | EWB: .050 (.091)  [-.146, .210]  SWB: .115 (.086)  [-.060, .276]  PWB: .096 (.083)  [-.087, .257] | **EWB: -.616 (.089)**  **[-.770, -.441]**  **SWB: -.584 (.084)**  **[-.735, -.413]**  **PWB: -.625 (.082)**  **[-.784, -.450]** | **EWB: -.570 (.033)**  **[-.629, -.490]**  **SWB: -.484 (.037)**  **[-.556, -.412]**  **PWB: -.547 (.032)**  **[-.607, -.480]** | EWB: .043 (.078)  [-.123, .179]  SWB: .098 (.074)  [-.050, .238]  PWB:.082 (.071)  [-.075, .220] |
|  |  |  |  |  |  |  |  |  |  | Unstandardised: | **.149 (.004)**  **[.142, .158]** | EWB: .027 (.051)  [-.078, .123]  SWB: .076 (.056)  [-.041, .174]  PWB: .065 (.057)  [-.058, .171] | **EWB: -.059 (.009)**  **[-.076, -.041]**  **SWB: -.067 (.010)**  **[-.086, -.047]**  **PWB: -.075 (.010)**  **[-.096, -.054]** | **EWB: -.055 (.004)**  **[-.064, -.047]**  **SWB: -.055 (.005)**  **[-.066, -.045]**  **PWB: -.065 (.005)**  **[-.077, -.056]** | EWB: .004 (.008)  [-.012, .018]  SWB: .011 (.008)  [-.006, .026]  PWB: .010 (.008)  [-.009, .026] |
| **Secondary Models** | | | | | | | | | | | | | | | |
| Anxiety | Measurement | 408 | 91 | 314 | .060 | .921 | .055 | - | - |  |  |  |  |  |  |
|  | Structural | 322 | 94 | - | .068 | .911 | - | .000 | EWB: **.346**  SWB: **.251**  PWB:**.366** | Standardised: | **.676 (.032)**  **[.607, .729]** | **EWB: -.337 (.073)**  **[-.472, -.195]**  **SWB: -.365 (.080)**  **[-.520, -.209]**  **PWB: -.424 (.075)**  **[-.520, -.209]** | **EWB: -.298 (.074)**  **[-.437, -.162]**  SWB: -.162 (.083)  [-.312, .005]  **PWB: -.226 (.073)**  **[-.370, -.087]** | **EWB: -.525 (.048)**  **[-.611, -.412]**  **SWB: -.410 (.055)**  **[-.503, -.300]**  **PWB: -.500 (.047)**  **[-.591, -.402]** | **EWB: -.226 (.051)**  **[-.321, -.129]**  **SWB: -.243 (.056)**  **[-.357, -.144]**  **PWB: -.284 (.053)**  **[-.386, -.177]** |
|  |  |  |  |  |  |  |  |  |  | Unstandardised: | **2.814 (.218)**  **[2.387, 3.221]** | **EWB: -.106 (.023)**  **[-.150, -.061]**  **SWB: -.135 (.030)**  **[-.197, -.077]**  **PWB: -.148 (.027)**  **[-.200, -.094]** | **EWB: -.386 (.102)**  **[-.571, -.209]**  SWB: -.254 (.131)  [-.511, .006]  **PWB: -.325 (.111)**  **[-.566, -.123]** | **EWB: -.680 (.082)**  **[-.857, -.539]**  **SWB: -.618 (.104)**  **[-.845, -.453]**  **PWB: -.725 (.084)**  **[-.911, -.567]** | **EWB: -.297 (.071)**  **[-.436, -.167]**  **SWB: -.375 (.093)**  **[-.583, -.213]**  **PWB: -.409 (.081)**  **[-.574, -.263]** |
| Depression | Measurement | 408 | 97 | 367 | .064 | .896 | .059 | - | - |  |  |  |  |  |  |
|  | Structural | 322 | 100 | - | .073 | .885 | - | .000 | EWB: **.421**  SWB: **.272**  PWB: **.430** | Standardised: | **.830 (.023)**  **[.775, .865]** | EWB: -.018 (.115)  [-.244, .215]  SWB: -.152 (.114)  [-.396, .051]  PWB: -.109 (.101)  [-.319, .098] | **EWB: -.628 (.107)**  **[-.843, -.412]**  **SWB: .379 (.113)**  **[-.592, -.152]**  **PWB: -.548 (.101)**  **[-.746, -.346]** | **EWB: -.642 (.041)**  **[-.722, -.563]**  **SWB: -.506 (.051)**  **[-.599, -.401]**  **PWB: -.635 (.042)**  **[-.719, -.549]** | EWB: -.015 (.096)  [-.192, .182]  SWB: -.125 (.095)  [-.322, .-042]  PWB: -.091 (.084)  [-.265, .081] |
|  |  |  |  |  |  |  |  |  |  | Unstandardised: | **4.563 (.471)**  **[3.668, 5.431]** | EWB: -.005 (.035)  [-.076, .065]  SWB: -.054 (.042)  [-.147, .018]  PWB: -.040 (.038)  [-.121, .036] | **EWB: -1.031 (.225)**  **[-1.532, -.661]**  **SWB: -.743 (.225)**  **[-1.217, -.282]**  **PWB: -1.120 (.241)**  **[-1.622, -.675]** | **EWB: -1.047 (.139)**  **[-1.360, -.840]**  **SWB: -1.005 (.132)**  **[-1.237, -.742]**  **PWB: -1.316 (.162)**  **[-1.598, -1.008]** | EWB: -.022 (.159)  [-.321, .305]  SWB: -.248 (.194)  [-.649, .082]  PWB: -.186 (.176)  [-.563, .160] |

*Note*. N = number of observations, DfM = model degrees of freedom, FP = Number of free parameters. EWB = Emotional Wellbeing, SWB = Social Wellbeing, PWB = Psychological Wellbeing.

For Bayesian estimation, Degrees of Freedom are not reported because the chi-square test that includes degrees of freedom are not used.

Round brackets = Posterior S.D. Square brackets = 95% credibility intervals.

Bolded = statistically significant, p<.05.

**Supplementary Table 3**

*Mplus Analysis Syntax for Each Main Model.*

| Model | Syntax | | |
| --- | --- | --- | --- |
|  | *Concurrent* | *Prospective* | *Prospective with WBT0 controlled* |
| **Primary** |  |  |  |
| GAD | MODEL:  ! measurement model  Anxiety by GADPhys GADUnc GADExc;  WBT0 by T0_EWB T0_SWB T0_PWB;  GADUnc WITH GADExc;  ! structural model  WBT0 on Anxiety;  GInt on Anxiety;  WBT0 on GInt;  Model indirect:  WBT0 IND Anxiety;  ANALYSIS:  Estimator = bayes;  OUTPUT:  Tech1 STDYX modindices TECH4; | MODEL:  ! measurement model  Anxiety by GADPhys GADUnc GADExc;  WBT1 by T1_EWB T1_SWB T1_PWB;  GADUnc WITH GADExc;  ! structural model  WBT1 on Anxiety;  GInt on Anxiety;  WBT1 on GInt;  Model indirect:  WBT1 IND Anxiety;  ANALYSIS: Estimator = BAYES;  OUTPUT:  Tech1 STDYX; | MODEL:  ! measurement model  Anxiety by GADPhys GADUnc GADExc;  WBT1 by T1_EWB T1_SWB T1_PWB;  WBT0 by T0_EWB T0_SWB T0_PWB;  T1_EWB WITH T0_EWB;  T1_SWB WITH T0_SWB;  T1_PWB WITH T0_PWB;  GADUnc WITH GADExc;  ! structural model  WBT1 on Anxiety WBT0;  GInt on Anxiety;  WBT1 on GInt;  Model indirect:  WBT1 IND Anxiety;  ANALYSIS: Estimator = BAYES;  OUTPUT: Tech1 STDYX; |
| MDD | MODEL:  ! measurement model  WBT0 by T0_EWB T0_SWB T0_PWB;  ! structural model  WBT0 on MDDSum;  MInt on MDDSum;  WBT0 on MInt;  Model indirect:  WBT0 IND MDDSum;  Analysis:  Estimator = Bayes;  OUTPUT:  Tech1 STDYX sampstat modindices TECH4; | MODEL:  ! measurement model  WBT1 by T1_EWB T1_SWB T1_PWB;  ! structural model  WBT1 on MDDSum;  MInt on MDDSum;  WBT1 on Mint;  Model indirect:  WBT1 IND MDDSum;  ANALYSIS: Estimator = BAYES;  OUTPUT:  Tech1 STDYX; | MODEL:  ! measurement model  WBT1 by T1_EWB T1_SWB T1_PWB;  WBT0 by T0_EWB T0_SWB T0_PWB;  T1_EWB WITH T0_EWB;  T1_SWB WITH T0_SWB;  T1_PWB WITH T0_PWB;  ! structural model  WBT1 on MDDSum WBT0;  MInt on MDDSum;  WBT1 on Mint;  Model indirect:  WBT1 IND MDDSum;  ANALYSIS: Estimator = BAYES;  OUTPUT: Tech1 STDYX; |
| PDD | MODEL:  ! measurement model  WBT0 by T0_EWB T0_SWB T0_PWB;  ! structural model  WBT0 on PDDSum;  PInt on PDDSum;  WBT0 on PInt;  Model indirect:  WBT0 IND PDDSum;  Analysis:  Estimator = Bayes;  OUTPUT:  Tech1 STDYX sampstat modindices TECH4; | MODEL:  ! measurement model  WBT1 by T1_EWB T1_SWB T1_PWB;  ! structural  WBT1 on PDDSum;  PInt on PDDSum;  WBT1 on PInt;  Model indirect:  WBT1 IND PDDSum;  ANALYSIS: Estimator = BAYES;  OUTPUT:  Tech1 STDYX; | MODEL:  ! measurement model  WBT1 by T1_EWB T1_SWB T1_PWB;  WBT0 by T0_EWB T0_SWB T0_PWB;  T1_EWB WITH T0_EWB;  T1_SWB WITH T0_SWB;  T1_PWB WITH T0_PWB;  ! structural model  WBT1 on PDDSum WBT0;  PInt on PDDSum;  WBT1 on PInt;  Model indirect:  WBT1 IND PDDSum;  ANALYSIS: Estimator = BAYES;  OUTPUT: Tech1 STDYX; |
| **Secondary** |  |  |  |
| Anxiety | MODEL:  !Measurement model  Anxiety BY AM1 AM2 AM3 AM4 AM5 AM6 AM7;  WBT0 BY T0_EWB T0_SWB T0_PWB;  SR_IMP BY WHOCog WHOMob WHOSelf WHOGet WHOLife WHOPar;  !Structural model  WBT0 ON Anxiety;  SR_IMP ON Anxiety;  WBT0 ON SR_IMP MedCondYN;  Model indirect:  WBT0 IND Anxiety;  ANALYSIS: Estimator = Bayes;  OUTPUT:  Tech1 STDYX modindices TECH4 sampstat; | MODEL:  !Measurement model  Anxiety BY AM1 AM2 AM3 AM4 AM5 AM6 AM7;  WBT1 BY T1_EWB T1_SWB T1_PWB;  SR_IMP BY WHOCog WHOMob WHOSelf WHOGet WHOLife WHOPar;  !Structural model  WBT1 ON Anxiety;  SR_IMP ON Anxiety;  WBT1 ON SR_IMP MedCondYN;  Model indirect:  WBT1 IND Anxiety;  ANALYSIS: Estimator = bayes;  OUTPUT:  Tech1 STDYX; | MODEL:  !Measurement model  Anxiety BY AM1 AM2 AM3 AM4 AM5 AM6 AM7;  WBT0 BY T0_EWB T0_SWB T0_PWB;  WBT1 BY T1_EWB T1_SWB T1_PWB;  SR_IMP BY WHOCog WHOMob WHOSelf WHOGet WHOLife WHOPar;  !Structural model  WBT1 ON Anxiety WBT0;  SR_IMP ON Anxiety;  WBT1 ON SR_IMP MedCondYN;  Model indirect:  WBT1 IND Anxiety;  ANALYSIS: Estimator = bayes; !change to MLR for measurement only  OUTPUT Tech1 STDYX; |
| Depression | MODEL:  !Measurement model  Depression BY DYS2 DYS3 DYS1 DYS4 DYS5 DYS6 DYS7 DYS8 DYS9;  WBT0 BY T0_EWB T0_SWB T0_PWB;  SR_IMP BY WHOCog WHOMob WHOSelf WHOGet WHOLife WHOPar;  !Structural model  WBT0 ON Depression;  SR_IMP ON Depression;  WBT0 ON SR_IMP MedCondYN;  Model indirect:  WBT0 IND Depression;  ANALYSIS: Estimator = Bayes;  OUTPUT:  Tech1 STDYX modindices TECH4 sampstat; | MODEL:  !Measurement model  Depression BY DYS2 DYS3 DYS1 DYS4 DYS5 DYS6 DYS7 DYS8 DYS9;  WBT1 BY T1_EWB T1_SWB T1_PWB;  SR_IMP BY WHOCog WHOMob WHOSelf WHOGet WHOLife WHOPar;  !Structural model  WBT1 ON Depression;  SR_IMP ON Depression;  WBT1 ON SR_IMP MedCondYN;  Model indirect:  WBT1 IND Depression;  ANALYSIS: Estimator = bayes;  OUTPUT:  Tech1 STDYX; | MODEL:  !Measurement model  Depression BY DYS2 DYS3 DYS1 DYS4 DYS5 DYS6 DYS7 DYS8 DYS9;  WBT1 BY T1_EWB T1_SWB T1_PWB;  WBT0 BY T0_EWB T0_SWB T0_PWB;  SR_IMP BY WHOCog WHOMob WHOSelf WHOGet WHOLife WHOPar;  !Structural model  WBT1 ON Depression WBT0;  SR_IMP ON Depression;  WBT1 ON SR_IMP MedCondYN;  Model indirect:  WBT1 IND Depression;  ANALYSIS: Estimator = bayes;  OUTPUT: Tech1 STDYX; |

**Supplementary Table 4**

*Pre-registered GAD exploratory model: Model fit statistics and standardised regression coefficients.*

| Variable | *a* path, estimate [95% CI]  (each symptom 🡪 impairment) | *b* path, estimate [95% CI]  (impairment 🡪 wellbeing) | *c’* Direct effect, estimate [95% CI]  (each symptom 🡪 wellbeing) | *c* Total effect, estimate [95% CI] | *ab* Indirect effect, estimate [95% CI] |
| --- | --- | --- | --- | --- | --- |
| Physical symptoms  Uncontrollable worrying  Excessive worrying | **.576 [.503, .661]**  **.190 [.070, .315]**  .092 [-.028, .216] | **-.253 [-.405, -.084]** | -.08 [-.258, .099]  **-.389 [-.582, -.193]**  **.222 [.027, .421]** | **-.227 [-.371, -.070]**  **-.439 [-.629, -.241]**  .199 [-.003, .403] | **-.147 [-.233, -.047]**  **-.045 [-.097, -.008]**  -.021 [-.064, .009] |

*Note.* Bolded = statistically significant, *p*<.05. RMSEA = .00, CFI = 1.00, PPP = .417, R^2^ = .235.

**Examination of Overlapping Item Measures**

Item overlap could be a potential concern when using these measures. Before commencing the study, authors carefully considered item overlap among the instruments (particularly the self-report measures) at the conceptual level, as described in more detail below. We found that majority of the items did not overlap across measures, and there were only small to moderate correlations among all measures. Taken together, concerns of measurement overlap were minimal.

MHC-SF and WHODAS - The closest related items were regarding 1. social and eudaimonic wellbeing with 2. social difficulties. Specifically, the MHC-SF has two items that ask about having warm and trusting relationships and belonging to a community, while the WHODAS has four items that assess difficulty in interacting with strangers, getting along with people, and maintaining and making friendships. Thus, it appeared that the items are all getting at components of socialising and relationships. The other items in each measure were considered distinct to one another conceptually. In the present study, the associations between these measures were small to moderate (r’s= -.41 to -.49).

MHC-SF and Symptom measures – The closest related items measure affect, with the MHC-SF assessing positive affect (how often did you feel happy?), and the depression symptom measures assessing negative affect (feeling down, depressed, hopeless) and anhedonia (lack of interest or pleasure). Positive affect and negative affect are considered distinct constructs within the literature (e.g., Schmukle et al., 2002). The other items do not overlap with one another conceptually. There were no concerns for the anxiety symptom items overlapping with wellbeing items. In the present study, the associations between these measures were small to moderate (r’s= -.27 to -.59).

Symptom measures and WHODAS - The cognitive impairment item in the WHODAS was somewhat similar to the cognition items in the IDAS and ADIS. For example, both ask about trouble concentrating, and IDAS asks about getting things done. However, the symptom measures assess psychopathology and perceived inefficacy, while the WHODAS measures disability resulting from this. The other items were not a concern. In the present study, the association between these measures was moderate (r’s=-.57 to -.64), with the exception of IDAS Dysphoria and WHODAS (r=-.74), perhaps reflecting the inclusion of the two similar cognition items.

**Anxiety and Related Disorders Interview Schedule for DSM-5 (ADIS-5) - Adult Version (Brown & Barlow, 2005)** **Scoring Details**

The GAD module assesses worries in nine life domains (e.g. school/work, health, finance, relationships), consisting of an item assessing excessiveness, and an item assessing uncontrollability, for each of the worry domains, as well as six items assessing physical symptoms, one item for impairment and one item for distress. The MDD and PDD modules consist of 12 and nine items assessing each symptom, respectively, one item for impairment, and one item for distress per module. The participant answered each item descriptively while the interviewer took notes and rated each item numerically (from 0, none, to 8, severe), based on severity and frequency.

The GAD module produces summed symptom composites for worry excessiveness, worry uncontrollability, and physical symptoms (e.g., Rutter & Brown, 2015). These composites were previously validated in 508 adult outpatients who presented for assessment and treatment of anxiety or mood symptoms (predominantly female (59%) and Caucasian (89%); mean age = 31.82, SD = 10.32, range 18 to 66) (Rutter & Brown, 2015). MDD and PDD symptoms were both summed across all items to produce dimensional composite scores for each disorder. The composite for MDD was previously validated in 700 adult outpatients who presented for treatment of anxiety or mood symptoms (60% female, 90.9% Caucasian, mean age = 32.38, SD = 11.62, range 18 to 74) (Brown & Naragon-Gainey, 2013). For exploratory analyses, the individual GAD, MDD and PDD symptom ratings were also used (composites for excessiveness and uncontrollability of worry, and single items for all others). For depressive symptoms where two items reflected one symptom (weight, sleep, appetite, psychomotor change), a single variable with the highest rated item was retained (e.g., weight gain and weight loss were combined into weight change). Impairment scores from GAD range from 0 to 8, whereas impairment scores from MDD and PDD were summed to form an overall impairment score for depression ranging from 0 to 16.

**Local Fit Information**

**Primary Models**

Local fit indicators suggested appropriate fit for most models (residuals correlations *r*s=-.000 to -.086 across anxiety models, *r*s=-.000 to -.093 across depression models). Notably, some residual correlations with wellbeing were >.100 (*r*s= -.253 to -.497) in the depression prospective models that did not control for prior wellbeing, suggesting those results should be interpreted with caution

**Secondary Models**

Local fit indicators suggested acceptable fit for anxiety models (residuals correlations rs= -.000 to -.090) and depression models (residuals correlations rs= -.002 to -.096), although some WHODAS subscales were slightly above .100. Residuals correlations were also slightly above .100 for wellbeing and two dysphoria items in prospective depression models.
